# The complete chloroplast genome sequence of *Elaeocarpus braceanus*: the first Celastraceae plastome

**DOI:** 10.1080/23802359.2020.1788444

**Published:** 2020-07-11

**Authors:** Qinghua Wang, Junwu Su, Yunqin Li, Xiaolong Yuan, Hong Zhou, Yi Wang

**Affiliations:** a Laboratory of Forest Plant Cultivation and Utilization, Yunnan Academy of Forestry & Grassland Science, Kunming, Yunnan, People's Republic of China; b Xishan Cooperative Forest Farm, Changning, Yunnan, China

**Keywords:** *Elaeocarpus braceanus*, chloroplast, Illumina sequencing, phylogenetic analysis

## Abstract

The first complete chloroplast genome (cpDNA) sequence of *Elaeocarpus braceanus* was determined from Illumina HiSeq pair-end sequencing data in this study. The cpDNA is 158,225 bp in length, contains a large single-copy region (LSC) of 85,731 bp and a small single-copy region (SSC) of 17,654 bp, which were separated by a pair of inverted repeats (IR) regions of 27,420 bp. The genome contains 133 genes, including 88 protein-coding genes, 8 ribosomal RNA genes, and 37 transfer RNA genes. Further phylogenomic analysis showed that *E. braceanus* clustered in a clade in Celastrales order.

*Elaeocarpus braceanus* is the species of the genus *Elaeocarpus* within the family Elaeocarpaceae. It is distributed in Yunnan, Tibet of China, Thailand, and India (Wang et al. 2015). It is a kind of wild fruit tree resource with Yunnan local ethnic flavor (Huang 1994; Dong 1995; Liu et al. 2016). There are many chemical components in *Elaeocarpus*, including alkaloids, terpenoids, flavonoids, coumarins, tannins and steroids. Some plants are used as traditional medicine to treat stress, depression, palpitation, liver disease, diabetes, and malaria (Wang et al. 2015). However, there has been no genomic study on *E. braceanus.*

Herein, we reported and characterized the complete *E. braceanus* plastid genome. The GenBank accession number is MT593043. One *E. braceanus* individual (specimen number: 2020011) was collected from Changning, Yunnan Province of China (24°55′66ʺN, 99°60′91ʺE). The specimen is stored at the Yunnan Academy of Forestry Herbarium, Kunming, China, and the accession number is WQH003. DNA was extracted from its fresh leaves using DNA Plantzol Reagent (Invitrogen, Carlsbad, CA, USA).

Paired-end reads were sequenced by using the Illumina HiSeq system (Illumina, San Diego, CA). In total, about 25.29 million high-quality clean reads were generated with trimmed adaptors. Aligning, assembly, and annotation were conducted by CLC *de novo* assembler (CLC Bio, Aarhus, Denmark), BLAST, GeSeq (Tillich et al. 2017), and GENEIOUS v 11.0.5 (Biomatters Ltd, Auckland, New Zealand), respectively. To confirm the phylogenetic position of *E. braceanus*, other five species of *Celastrales* order from NCBI were aligned using MAFFT v.7 (Katoh and Standley 2013). The Auto algorithm in the MAFFT alignment software was used to align the eight complete genome sequences and the G-INS-i algorithm was used to align the partial complex sequences. The maximum likelihood (ML) bootstrap analysis was conducted using RAxML (Stamatakis 2006); bootstrap probability values were calculated from 1000 replicates. *Salacia amplifolia* (MK799641) and *Monimopetalum chinense* (MK450440) were served as the outgroup.

The complete *E. braceanus* plastid genome is a circular DNA molecule with the length of 158,225 bp, containing a large single-copy region (LSC) of 85,731 bp and a small single-copy region (SSC) of 17,654 bp, which were separated by a pair of inverted repeats (IR) regions of 27,420 bp. The overall GC content of the whole genome is 37.1%, and the corresponding values of the LSC, SSC, and IR regions are 35.0%, 31.2%, and 42.2%, respectively. The plastid genome contained 133 genes, including 88 protein-coding genes, 8 ribosomal RNA genes, and 37 transfer RNA genes. Phylogenetic analysis showed that *E. braceanus* clustered in a unique clade in *Celastrales* order (Figure 1). The determination of the complete plastid genome sequences provided new molecular data to illuminate the *Celastrales* order evolution.

**Figure 1.**
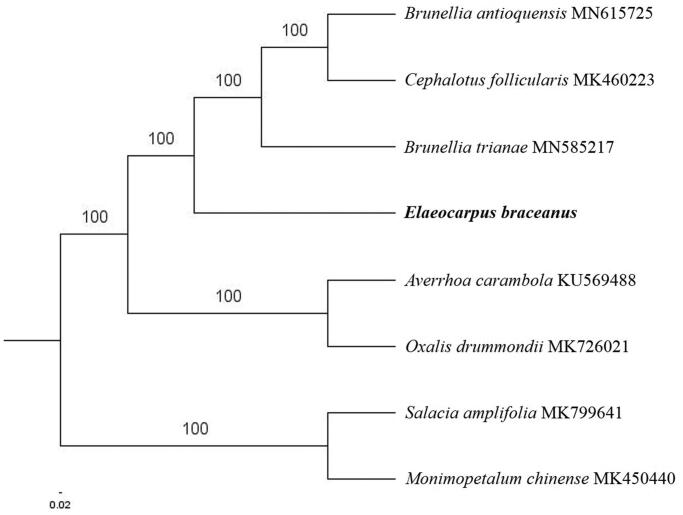
The maximum-likelihood tree based on the six chloroplast genomes of order *Celastrales*. The bootstrap value based on 1000 replicates is shown on each node.

## Data Availability

The data that support the findings of this study are openly available in NCBI GenBank database at (https://www.ncbi.nlm.nih.gov) with the accession number is MT593043, which permits unrestricted use, distribution, and reproduction in any medium, provided the original work is properly cited.
